# Ferroelectric Properties of Polymer–Semiconductor Hybrid Material or Composite under Optical Excitation

**DOI:** 10.3390/polym16070929

**Published:** 2024-03-28

**Authors:** Michael Kober, David Smykalla, Bernd Ploss, Maria Wächtler, Krishan Kumar, Michael Stelter, Sebastian Engel

**Affiliations:** 1 Institute for Technical and Environmental Chemistry, Friedrich Schiller University Jena, Philosophenweg 7a, 07743 Jena, Germany; david.smykalla@uni-jena.de (D.S.); michael.stelter@uni-jena.de (M.S.); sebastian.engel@uni-jena.de (S.E.); 2 Department of SciTec, University of Applied Sciences Jena, Carl-Zeiss-Promenade 2, 07745 Jena, Germany; bernd.ploss@eah-jena.de; 3 Chemistry Department and State Research Center OPTIMAS, RPTU Kaiserslautern-Landau, Erwin-Schrödinger-Str. 52, 67663 Kaiserslautern, Germany; maria.waechtler@chem.rptu.de; 4 Department Functional Interfaces, Leibniz Institute of Photonic Technology Jena, Albert-Einstein-Straße 9, 07745 Jena, Germany; kumar.krishan@leibniz-ipht.de

**Keywords:** semiconductor (micro)particle–ferroelectric polymer matrix hybrid material, quantum dots, photoconductivity, optical excitation

## Abstract

Polymer–semiconductor hybrid materials or composites have been investigated with respect to their microstructure, optical, photoconductive, and ferroelectric properties. For this purpose, either CdSe quantum dots or (Cd:Zn)S microparticles were dispersed in poly(vinylidenefluoride-trifluoroethylene) solution and hot pressed to films. In both material systems, the electrical conductivity and the polarization behavior could be controlled by the intensity of the optical excitation. The simultaneous high optical transparency of the CdSe quantum-dot-based hybrid materials makes them particularly interesting for applications in the field of flexible, high-resolution sensors.

## 1. Introduction

Ferroelectric polymers such as poly(vinylidenefluoride-trifluoroethylene) [P(VDF-TrFE)] are characterized by their mechanical flexibility, lightness, conformability, and facile processability compared to classic ceramic ferroelectrics such as lead zirconate titanate [PZT] and BaTiO_3_. These favorable features make them candidates for applications in medicine, energy harvesting, and data storage, as well as actuator and sensor technologies [1,2,3,4,5,6].

The ferroelectric properties of the semicrystalline P(VDF-TrFE) depend mainly on its polar, crystalline β-phase and its interaction with the amorphous phase [7,8,9,10,11]. Beyond that, their electrical and ferroelectric behavior can be tailored by dispersing particles into the polymer matrix [12,13,14,15]. In this way 0–3 composites of ferroelectric particles and a ferroelectric matrix can be utilized to produce sensors that respond selectively to pressure or temperature [16]. For their use as such bifunctional sensors, a selective poling of both constituents, particles and matrix, is necessary, which requires an increase in the matrix conductivity [16,17]. Currently, this is achieved by increasing the temperature or doping of the composite [17,18,19]. However, these methods are limiting the spatial resolution of the sensor elements. In order to overcome this limitation, the electrical and ferroelectric properties of the composite have to be controlled via an external high-resolution trigger, such as light acting on (photoconductive) semiconductor particles, which are additionally dispersed within the polymer matrix.

It is well known that photoexcitation results in an increasing electrical conductivity at composites of (Cd:Zn)S semiconductor particles and P(VDF-TrFE) [20]. Poling these composites under optical excitation with light of a suitable wavelength is also associated with an increase in polarization [20,21]. However, these composites exhibit an inhomogeneous polarization distribution due to the strongly decreasing light intensity profile over the sample thickness. This in turn is due to the high concentration of particles in the transparent polymer, which is necessary for an optimum response to the optical excitation of the system [21]. This limits the potential for future applications enormously [20,21]. In addition, various fields of application, such as robotics, require optically transparent sensors and actuators.

Therefore, the aim of this work is the development of a hybrid material where both the electrical conductivity and the polarization behavior are optically controllable and homogeneous, combined with high transparency at the same time.

For this purpose, films of CdSe quantum dots (QDs) in a P(VDF-TrFE) matrix were investigated as a novel hybrid material in comparison to a composite system consisting of microscale (Cd:Zn)S particles in P(VDF-TrFE). The simultaneous characterization of the different properties such as microstructure, transparency, conductivity, and ferroelectricity results in a better understanding of the mutual influence of these parameters on each other.

## 2. Materials and Methods

### 2.1. Sample Preparation

**Figure 1:**
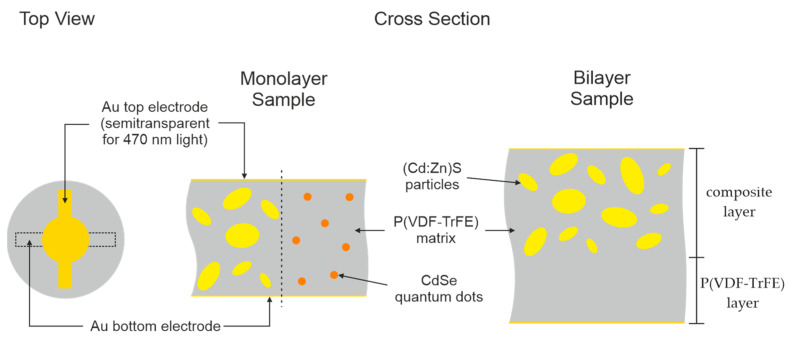
Schematic structure of (Cd:Zn)S composite and CdSe hybrid material samples, as well as bilayer (Cd:Zn)S composite//P(VDF-TrFE) samples.

Composites and hybrid materials of P(VDF-30TrFE) (Piezotech Arkema, Pierre-Benite Cedex, France) and either (Cd:Zn)S microparticles or CdSe QDs (denoted as filler) were fabricated at specific particle concentrations between 0 vol% and 15 vol% or 0 vol% and 0.2 vol%, respectively. In the case of the (Cd:Zn)S/P(VDF-TrFE) composites, (Cd:Zn)S powder with a mean particle diameter of 500 nm (Kremer Pigments, Aichstetten, Germany) was dispersed in 1.5 g of 10 wt% P(VDF-TrFE)/N,N-dimethylformamide [DMF] solution using magnetic stirring (10 min) first and an ultrasonic sonotrode (5 min) second. Regarding the CdSe/P(VDF-TrFE) hybrid material, self-synthesized 2.5 nm CdSe QDs (Leibniz Institute for Photonic Technologies, Jena, Germany) functionalized with 1-methoxy-2-propyl acetate [MPA] and separated in N-methylformamide [NMF] were dispersed in 1.5 g of 10 wt% P(VDF-TrFE)/NMF solution by magnetic stirring for 10 min. To calculate the necessary component amounts, the size of the QDs as well as their concentration in NMF has been taken into account. Furthermore, polymer or (Cd:Zn)S densities of 1.9 g/cm^3^ [22] and 4.82 g/cm^3^ [23] were assumed. For solvent evaporation, the dispersions were transferred to Petri dishes and in the case of the NMF containing dispersions additionally stored under reduced pressure. Flexible films with a thickness of (21 ± 2) µm and a diameter of about 10 mm were produced from the composites and hybrid materials by hot pressing for 3 min at 180 °C and a pressing force of 40 kN. In the case of the (Cd:Zn)S composites, two layers each initially pressed to about 10 µm in thickness were pressed together to prevent short circuits during poling caused by particle agglomerates. To further increase stability against short circuits, bilayer (Cd:Zn)S composite//P(VDF-TrFE) samples of (31 ± 2) µm in thickness were produced by pressing together a layer of 10 vol% (Cd:Zn)S composite (thickness 20 µm) and a pure polymer layer (Figure 1). For a cooling process within a few seconds in air, the films were surrounded by 10 µm-thick steel sheets to realize a simple transfer out of the press. After removing the steel sheets, the films were annealed for 1.5 h at 130 °C to ensure both a constant crystallinity of the polymer and to reduce mechanical stresses. Finally, Au electrodes with a diameter of 5 mm and additional contact fingers were sputtered onto both sides of the films. The thickness was 30 nm on the top and 60 nm on the bottom side (Figure 1). For the 30 nm-thick top electrode, a transmittance of 14% was determined at the utilized optical excitation wavelength of 470 nm. The resistance between the two adjacent contact fingers of the front electrode was low (about 4 Ω) compared to the minimum DC resistance, measured between the top and bottom electrode (at least about 1 GΩ depending on composition of the composite and its optical excitation intensity).

### 2.2. Microstructure and Optical Characterization

The crystallinity X_β_ of P(VDF-TrFE) from the hot pressed and annealed samples was examined by an X-ray diffractometer [XRD] (D8 Advance, Bruker AXS, Karlsruhe, Germany) using CuKα-radiation at a 40 kV operating voltage and 40 mA current. The scanning speed was 0.5 s/step and the step size 0.03° for the 2θ range from 10° to 60°. The particle distribution in the polymer matrix was investigated by means of optical (VHX 7000, Keyence, Neu-Isenburg, Germany) and scanning electron microscopy (SEM, Ultra 55, Zeiss Microscopy, Jena, Germany). The SEM characterization was carried out at an accelerating voltage of 5 kV on blade-cut sample cross sections, which were deposited with a 2 nm-thick Au layer for this purpose. In addition, the particle size distribution of the (Cd:Zn)S powder was determined by laser beam diffraction (Mastersizer 2000 with Hydro2000, Malvern Panalytical, Kassel, Germany) utilizing the Fraunhofer method. For this purpose, the powder was dispersed in a 0.2 wt% sodium pyrophosphate solution by various dispersion methods (stirring by spatula, magnetic stirring, magnetic stirring and ultrasonic sonotrode).

The transmittance of pure P(VDF-TrFE) as well as the composite and hybrid samples of various thickness (without electrodes) was measured at a wavelength of 470 nm, the same as for optical excitation. For this purpose, a setup consisting of the utilized LED (LXML-PB01-0023, LUMILEDS, Amsterdam, The Netherlands; 470 nm central wavelength and 20 nm FWHM), collimator lenses, iris aperture, and optical power detector (S130VC, Thorlabs, Dachau/München, Germany) was used. The transmittance was determined from the measured radiant power with and without a sample in the beam path. The hot-pressed samples with a thickness between 10 µm and 36 µm were positioned between two glass slides during the measurement to prevent them from bending. To measure the transmittance at lower sample thicknesses, polymer and composite or hybrid films were also produced by spin coating on a glass slide. UV–Vis spectroscopy (V-780 spectrophotometer, Jasco Inc., Easton, PA, USA) was performed for measuring the absorption spectra of the CdSe QDs and the transmittance spectra of P(VDF-TrFE) and CdSe-QDs hybrid material films.

### 2.3. Electrical and Ferroelectric Properties

The current [*I*]-electrical field strength [*E*] curves and polarization hysteresis of the samples were characterized under optical excitation conditions with different illumination intensities. Optical excitation was provided by an LED that illuminated the semi-transparent top electrode. The illumination intensity at the top electrode was calibrated with an optical power detector. The *I-E*-curves were recorded by a source measure unit (4100, Keithley, Solon, OH, USA). Field strength amplitudes of 10 MV/m were used for the measurements, whereby the field strength was changed in steps of 1 MV/m starting from 0 MV/m. The polarization hysteresis was characterized with a setup described elsewhere [20]. Poling was performed in each case using a triangular voltage with field strength amplitudes up to 70 MV/m and frequencies *f* between 1 Hz and 100 Hz. The remanent polarization *P_r_* was determined from the hysteresis after 2 min poling time. The pyroelectric coefficient *p* of the poled samples was measured using the harmonic waveform technique [HWT] [24]. For this purpose, the pyroelectric current was recorded while the sample temperature *T*(*t*) was sinusoidally modulated with a frequency of 10 mHz using a temperature amplitude of 2 K and an average temperature of 25 °C. For the relaxation of excess charges, short-circuits were applied for a period of 30 min between sample polarization and HWT measurement. Depolarization heat treatment between each series of measurement was performed by heating the samples above the Currie temperature of the polymer (*T_c_* ≈ 100 °C) to 130 °C for 5 min, using a constant heating and cooling rate of 220 K/h while ensuring a short-circuit condition between the top and bottom electrode. Complete depolarization was confirmed by HWT measurement.

## 3. Results

### 3.1. Composite Morphology

**Figure 2:**
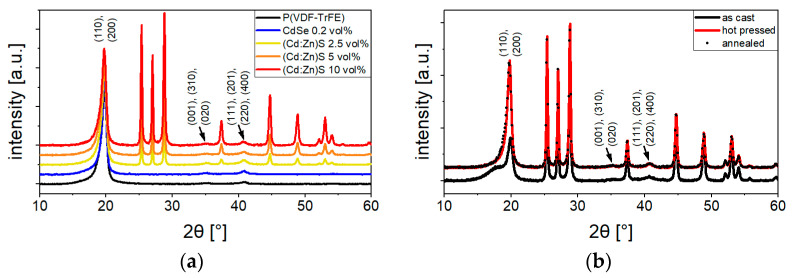
(**a**) XRD diffractograms of composites and hybrid material of different filler concentrations after annealing. (**b**) Diffractograms of the 10 vol% (Cd:Zn)S composite after different process steps normalized using the particle signals. A slight shift in the superimposed (110)/(200) β-peaks in the hot-pressed and annealed composite occurred compared to the sample after solvent evaporation (as cast). This is attributed to an increase in polymer chain distances due to additional gauche defects introduced into the β-phase during its transition from the paraelectric to the ferroelectric phase during cooling after hot pressing [26]. The indexing of the β-phase was performed according to literature data [27,28,29], whereas all peaks without indexes belong to the (Cd:Zn)S particles which was ensured through measurements with the utilized (Cd:Zn)S powder.

The indexed diffractograms of the composites and hybrid films after annealing (Figure 2a) show the presence of the amorphous and β-phase in the P(VDF-TrFE) matrix and of the (Cd:Zn)S particles (all peaks without indexing can be attributed to the (Cd:Zn)S particles). No signal of the CdSe QDs appeared even at the highest used concentration of 0.2 vol%. The cumulative peak in the vicinity of 2θ = 19.9° was decomposed into its amorphous (area *A_a_*) and crystalline fractions (area *A_β_*) using a pseudo-Voigt function to determine the crystallinity *X_β_* of the polymer using the method of Tajitsu et al. [25] according to Equation (1):

(1) $$X_{\beta }\left[vol\%\right]=\frac{A_{\beta }}{A_{\beta }+A_{a}}.$$

Regardless of the particle or QDs concentration, the fraction of ferroelectrically active β-phase in the polymer (equivalent to its crystallinity) is about 66 vol% for all composites, hybrid materials, and pure P(VDF-TrFE). The total β-phase fraction in the samples differs therefore depending on their filler concentration (e.g., 66 vol% for the 0.2 vol% CdSe hybrid material or 59 vol% for the 10 vol% (Cd:Zn)S composite). Using the 10 vol% (Cd:Zn)S composite as an example, Figure 2b shows that the β-phase fraction of the samples, which is low after evaporation of the solvent (as cast), had already reached its maximum after hot pressing. The subsequent annealing did not lead to any measurable increase in the β-phase content. This also rules out the possibility that the depolarization heat treatments between the series of experiments led to a change in the polarization behavior by changing the amount of β-phase.

**Figure 3:**
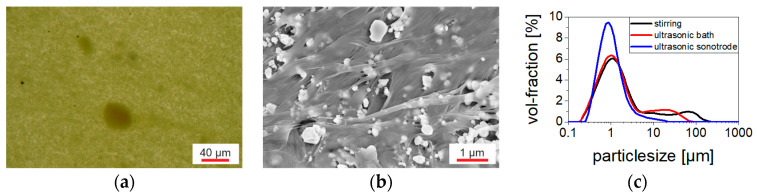
(**a**) 2.5 vol% (Cd:Zn)S composite (transmitted optical microscope) with darker particles and particle agglomerates within the transparent polymer matrix. (**b**) 10 vol% (Cd:Zn)S composite (SEM) with particles and particle agglomerates within the polymer matrix. (**c**) Particle size distribution (laser beam diffraction) of (Cd:Zn)S powder in 0.2 wt% sodium pyrophosphate solution after dispersion by different techniques (stirring with spatula, combined magnetic stirring and ultrasonic bath treatment, ultrasonic sonotrode treatment).

The morphology studies performed at the (Cd:Zn)S composites by optical microscope and SEM (Figure 3a,b) show a wide particle distribution of primary particles with diameters < 0.1 µm and agglomerates > 20 µm (20 µm corresponds to the sample thickness of monolayer composite samples) within the polymer matrix. Thus, the ultrasonic sonotrode used for particle separation during composite preparation did not cause complete destruction of the (Cd:Zn)S agglomerates. However, compared to mere stirring with a spatula or combined magnetic stirring and ultrasonic bath treatment, the increased energy input reached by the sonotrode resulted in a finer particle distribution (Figure 3c). In particular, it reduced the proportion of particles with diameters equal to or larger than the sample thickness, which were the main cause for short-circuits during poling.

**Figure 4:**
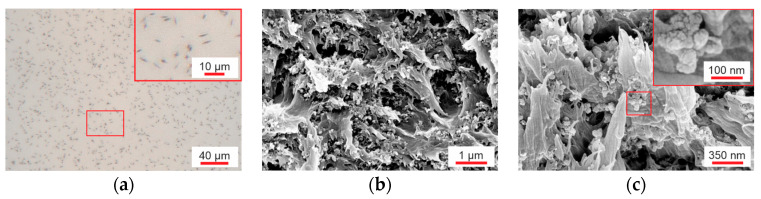
(**a**) Particle distribution in the 0.2 vol% CdSe hybrid material (transmitted optical microscope) showing dark, rod-shaped CdSe QDs accumulations in the polymer matrix, which appears slightly reddish in color due to the additional fraction of CdSe clusters distributed in it. (**b**,**c**) 0.2 vol% CdSe hybrid material (SEM) showing clusters each formed by multiple CdSe QDs within the polymer matrix.

In the CdSe hybrid material, rod-shaped particle accumulations were formed by separation of the P(VDF-TrFE) solution and QDs (Figure 4a). However, some of the CdSe QDs are additionally distributed in the polymer matrix (Figure 4b,c), where they form clusters each consisting of several QDs with sizes up to 100 nm. Nevertheless, the CdSe hybrid materials exhibit a finer particle distribution compared to the (Cd:Zn)S composites, which is clearly shown by the comparison of the SEM images (Figure 3b and Figure 4b).

### 3.2. Electrical Properties

**Figure 5:**
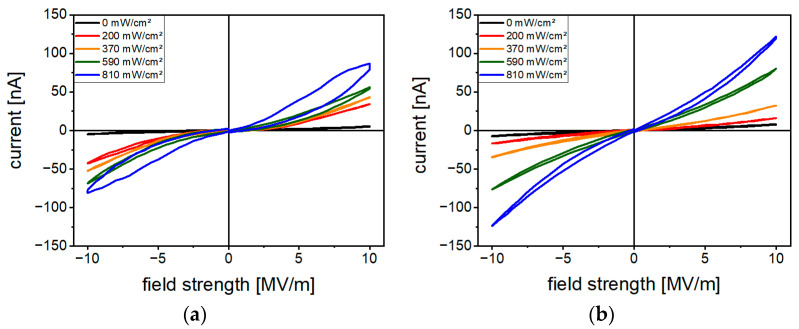
*I-E* curves at different optical excitation intensities for (**a**) a 10 vol% (Cd:Zn)S composite and (**b**) a 0.2 vol% CdSe hybrid material.

Figure 5a,b show the *I-E* curves measured at different optical excitation intensities for the most intensively studied samples containing either 10 vol% (Cd:Zn)S or 0.2 vol% CdSe. They exhibit a non-linear *I-E* dependence. In addition, the *I-E* curves show a hysteresis effect, which is especially pronounced in case of the 10 vol% (Cd:Zn)S composite under the highest optical excitation intensities.

**Figure 6:**
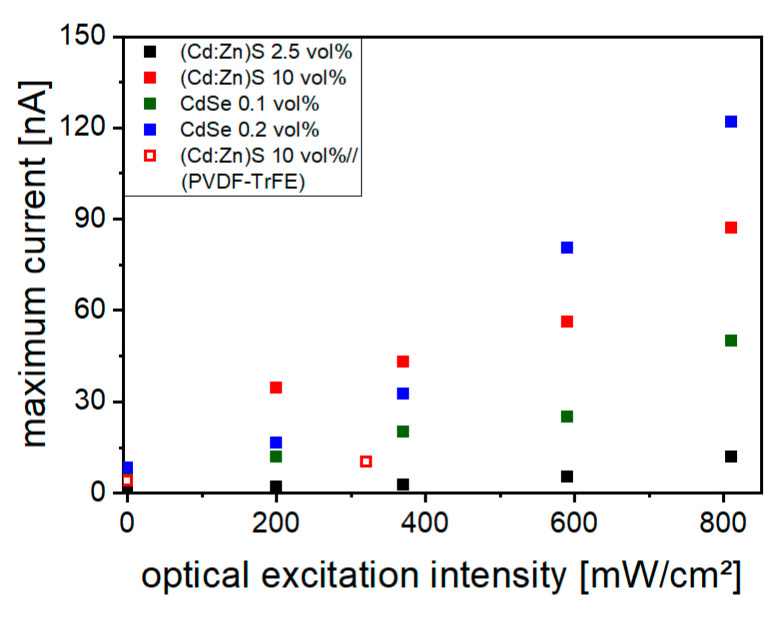
Maximum *I* determined from the *I-E* curves measured at different optical excitation intensities for composites and hybrid materials with different filler concentrations and a bilayer 10 vol% (Cd:Zn)S composite//P(VDF-TrFE) sample.

The maximum currents determined from each of these curves for different filler concentrations are presented in Figure 6. They show a gradual increase in the electrical conductivity with increasing illumination intensity. This is attributed to exciton generation in the particles and QDs by photoexcitation (see also Section 3.3 Optical Properties) and the associated increase in conductivity of the semiconductor filler phase. The influence of the CdSe QDs on the optically controllable conductivity is more pronounced than that of the (Cd:Zn)S particles. A concentration of 0.1 vol% CdSe already leads to a higher conductivity than a concentration of 2.5 vol% (Cd:Zn)S. At the highest used excitation intensities of 810 W/cm^2^, an increase in conductivity by a factor of 15 (0.2 vol% CdSe) and 10 (10 vol% (Cd:Zn)S) was measured compared to pure P(VDF-TrFE), which has about 130 GΩ DC resistance at 10 MV/m. At illumination intensities between 380 mW/cm^2^ and 590 mW/cm^2^, which were used for optical excitation during poling, the 10 vol% (Cd:Zn)S composite and the 0.2 vol% CdSe hybrid material show similar conductivities.

Even without optical excitation, particles and QDs led to a tripling (0.2 vol% CdSe) or doubling (10 vol% (Cd:Zn)S) of the electrical conductivity compared to pure P(VDF-TrFE). In contrast, the use of an additional polymer layer, as for the bilayer 10 vol% (Cd:Zn)S composite//P(VDF-TrFE) samples, led to a significant reduction in conductivity (Figure 6). In addition, conductivity measurements were performed on 10 vol% C(Cd:Zn)S composites, which were produced in a different way compared to the description in Section 2.1. An ultrasonic bath was used for the purpose of (Cd:Zn)S particle singulation instead of a sonotrode. Furthermore, those composites were prepared by a single hot-pressing step (not by pressing together two layers each initially pressed to about 10 µm in thickness) and are therefore referred to as “single pressed” 10 vol% (Cd:Zn)S composite samples. The electrical conductivity of those “single pressed” 10 vol% (Cd:Zn)S composites measured without and with optical excitation was approximately twice as high as that of the other 10 vol% (Cd:Zn)S composites.

### 3.3. Optical Properties

**Figure 7:**
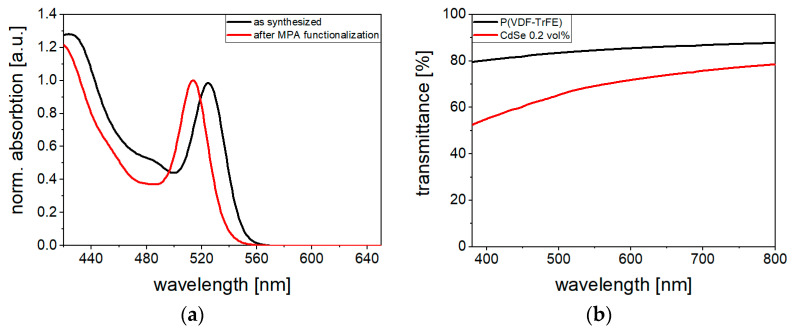
(**a**) Normalized absorption spectra of CdSe QDs before and after functionalization of the surface with MPA. The observed blue shift after functionalization is attributed to a removal of surface cations decreasing the effective core size of the quantum dots [31,32]. (**b**) Transmittance spectra of 20 µm-thick P(VDF-TrFE) and 0.2 vol% CdSe hybrid material film.

The UV–Vis spectra of the CdSe QDs in solution before and after MPA functionalization (Figure 7a) show the onset of absorption due to beginning photoexcitation at a wavelength of about 560 nm. This is accompanied by the generation of additional charge carriers. In the UV–Vis transmittance spectrum of the 0.2 vol% CdSe hybrid material film (Figure 7b), the onset of photoexcitation is not evident due to the low QDs concentration. In the UV–Vis spectra measured elsewhere [20] on 30 µm-thick 10 vol% (Cd:Zn)S composite films (using the same (Cd:Zn)S particles as in the samples measured here), the onset of absorption was detected at wavelengths of 490 nm. This could be attributed the band gap of (Cd:Zn)S [30].

**Figure 8:**
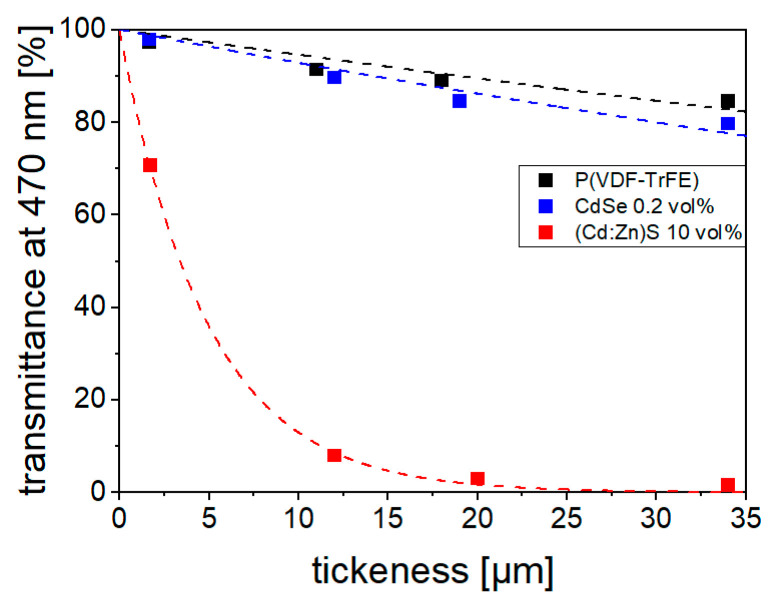
Transmittance *T*_470_ of P(VDF-TrFE), 0.2 vol% CdSe hybrid material, and 10 vol% (Cd:Zn)S composite for different film thicknesses at a wavelength of 470 nm. The light intensity profiles *J*(*d*) as a function of sample depth *d* were estimated using Lambert Beer’s law.

The transmittance *T*_470_ at a wavelength of 470 nm, which was also used for optical excitation during poling, is shown in Figure 8. In the 10 vol% (Cd:Zn)S composites, almost complete absorption occurs at a 20 µm film thickness. In contrast, the *T*_470_ of the 0.2 vol% CdSe hybrid material is only slightly lower than that of the pure polymer (*T*_470_ = 89% at 18 µm thickness) due its low filler concentration. There is a difference in the *T* values of the 0.2 vol% composite in the UV–Vis measurement (Figure 7b) and the measurement at the experimental wavelength of 470 nm (Figure 8). This is attributed to the effect of surface reflectance of the two samples used and the influence of sample bending on transmittance (glass slides to prevent bending could not be implemented in the UV–Vis setup as for the *T*_470_ setup). In Figure 8, a rough estimate of the sample depth [*d*]-dependent light intensity profiles *J*(*d*) was made using Lambert Beer’s law. Due to its higher transmittance, the light intensity profile in the 0.2 vol% CdSe hybrid material shows a significantly increased homogeneity compared to the 10 vol% (Cd:Zn)S composite. As will be explained later, it is assumed that this influences charge carrier distribution and polarization behavior.

**Figure 9:**
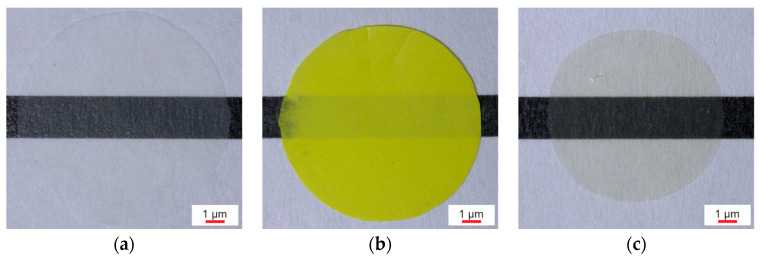
Appearance of 20 µm thick films of (**a**) pure P(VDF-TrFE), (**b**) a 10 vol% (Cd:Zn)S particle composite, and (**c**) a 0.2 vol% CdSe QDs hybrid material.

The effects of the different filler concentrations and types on the visual appearance of the composites and hybrid materials are illustrated in Figure 9. While the 0.2 vol% CdSe hybrid material shows a faint reddish discoloration compared to the colorless P(VDF-TrFE), the 10 vol% (Cd.Zn)S composite has taken on the yellow color of the particles.

### 3.4. Ferroelectric Properties

#### 3.4.1. Influence of Particle Concentration

**Figure 10:**
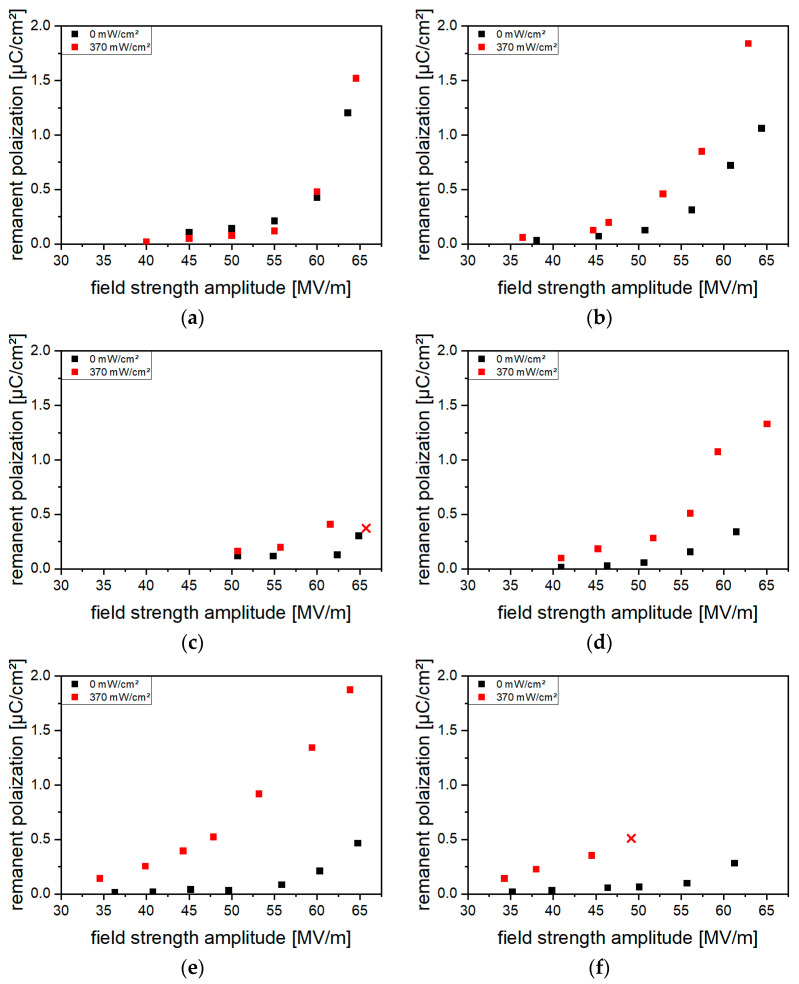
Remanent polarization *P_r_* of the (Cd:Zn)S composites for particle concentrations of (**a**) 1 vol%, (**b**) 2.5 vol%, (**c**) 5 vol%, (**d**) 7.5 vol%, (**e**) 10 vol%, and (**f**) 15 vol% without and with optical excitation during poling. Poling was performed at frequencies of 10 Hz with a stepwise increase in the field strength amplitude. After the measurement without optical excitation, a depolarization heat treatment was performed before the measurement was repeated with optical excitation during poling. At measuring points marked with an ×, the sample was destructed through electric breakdown.

**Figure 11:**
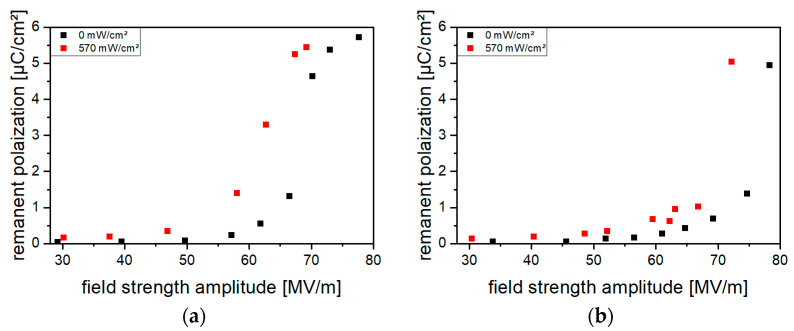
Remanent polarization *P_r_* of CdSe hybrid materials for QDs concentrations of (**a**) 0.1 vol% and (**b**) 0.2 vol% without and with optical excitation during poling. Poling was performed at frequencies of 10 Hz with a stepwise increase in the field strength amplitude. After the measurement without optical excitation, a depolarization heat treatment was performed before the measurement was repeated with optical excitation during poling.

As Figure 10 shows for the (Cd:Zn)S composites, an increase in *P_r_* by optical excitation during poling occurred at particle concentrations of ≥2.5 vol%. The effect was more pronounced with rising particle amounts and highest for particle concentrations of 10 vol% and 15 vol%. However, the higher the particle concentration, the more electrical breakdowns occurred in the composites during poling. Increasing the optical excitation intensity also led to more breakdowns; thus, no intensities above 370 mW/cm^2^ were used. For the CdSe hybrid materials (Figure 11), an increase in *P_r_* upon poling under optical excitation already occurred at QDs concentrations of ≥0.1 vol%. Furthermore, a higher breakdown stability enabled excitation intensities of 570 mW/cm^2^. Nevertheless, at CdSe concentrations > 0.2 vol%, polarization under optical excitation with light intensities of 570 mW/cm^2^ was not applicable due to electrical breakdown.

One aim of further investigations was to maximize the raise of remanent polarization by optical excitation during poling. For this purpose, the most suitable filler concentrations were selected. In the case of the (Cd:Zn)S composites, the concentration of 10 vol% was chosen because of its lower breakdown sensitivity compared to the concentration of 15 vol%. In addition, breakdowns could be prevented using 10 vol% (Cd:Zn)S composite//P(VDF-TrFE) bilayer samples. For the CdSe hybrid materials, a QDs concentration of 0.2 vol% was selected for further investigations. It showed a more pronounced increase in *P_r_* compared to the concentration of 0.1 vol% at the polarization frequency of *f* = 1 Hz mainly used later. In addition, due to their similar conductivity under optical excitation intensities used for poling (Figure 6), the two filler concentrations of 10 vol% (Cd:Zn)S and 0.2 vol% CdSe were suitable for a comparative analysis.

#### 3.4.2. Influence of Poling Frequency

The influence of poling frequency on the polarization behavior of the composites and hybrid materials under optical excitation was investigated starting from 10 Hz. This value has already been used in previous studies on (Cd:Zn)S composites [20,21] and is well below the frequency range of ≥1 kHz, in which an increase in *P_r_* with decreasing frequency occurs during electrode poling of pure P(VDF-TrFE) [33].

**Figure 12:**
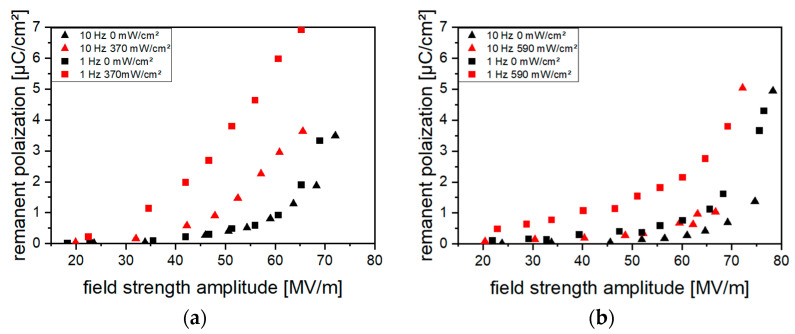
Remanent polarization *P_r_* without and with optical excitation during poling at poling frequencies of 10 Hz and 1 Hz for samples containing (**a**) 10 vol% (Cd:Zn)S and (**b**) 0.2 vol% CdSe. Poling at each frequency was performed with a stepwise increase in the field strength amplitude. Between each measuring series with a distinct frequency and excitation intensity, a depolarization heat treatment was performed.

Frequencies higher than 10 Hz led to sample destruction due to electrical breakdown already occurring at low field strength amplitudes, in particular if poling was performed under optical excitation. This is attributed to an increase in energy dissipated into heat during the reversal of polarity (reversal losses). If poling was performed under optical excitation, a frequency decrease from 10 Hz to 1 Hz caused a reduction in the field strength amplitude necessary for polarization and it led to an increase in *P_r_* (Figure 12). Additionally, the difference between *P_r_* reached through poling with and without optical excitation increased.

#### 3.4.3. Influence of Poling Field Strength Amplitude

**Figure 13:**
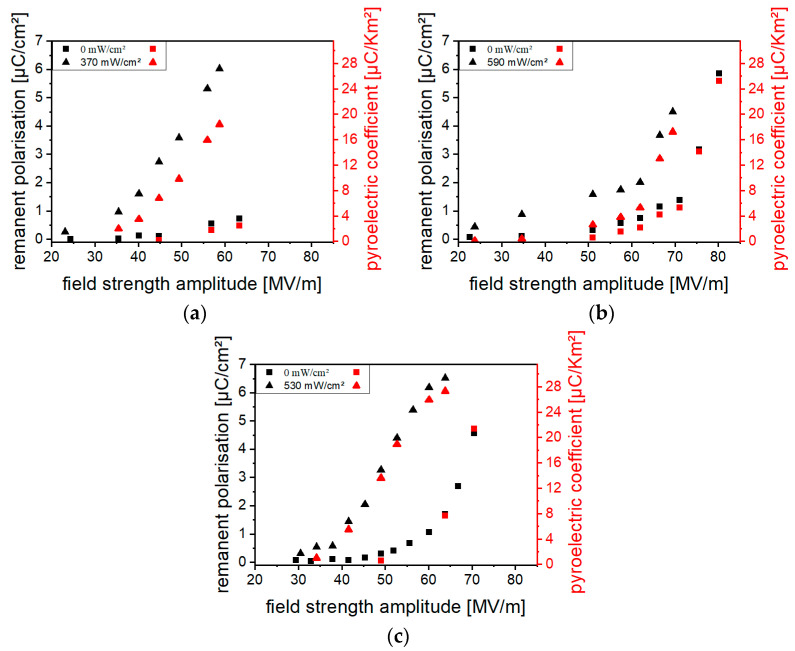
Remanent polarization *P_r_* and the pyroelectric coefficient *p* at different field strength amplitudes without and with optical excitation during poling for (**a**) a 10 vol% (Cd:Zn)S composite, (**b**) a 0.2 vol% CdSe hybrid material, and (**c**) a bilayer 10 vol% (Cd:Zn)S composite//P(VDF-TrFE) sample. Poling was performed at frequencies of 1 Hz with a stepwise increase in the field strength amplitude. After the measurement without optical excitation, a depolarization heat treatment was performed before the measurement was repeated with optical excitation during poling.

The increases in *P_r_* and *p* through optical excitation for a composite with 10 vol% (Cd:Zn)S and a hybrid material with 0.2 vol% CdSe, as well as for a 10 vol% (Cd:Zn)S composite//P(VDF-TrFE) bilayer sample, are shown in Figure 13. The absolute difference in polarization reached with and without optical excitation during poling varies with the field strength amplitude. The maximum absolute difference occurred at field strengths between 55 MV/m and 70 MV/m, at which slight polarization is measured even without optical excitation during poling. The polarization increase due to illumination was the highest for the composites with (Cd:Zn)S. For example, *P_r_* was 1 µC/cm^2^ without and 6 µC/cm^2^ with optical excitation at 60 MV/m for the bilayer sample. In contrast to the (Cd:Zn)S monolayer composites, no breakdowns occurred in the bilayer samples even at the highest field strength amplitudes or excitation intensities used. Furthermore, it can be seen from Figure 13 that the optical excitation during poling decreases the minimum field strength amplitude necessary for polarization. Regarding *p*, for instance, polarization occurred in the bilayer sample at field strength amplitudes < 40 MV/m with optical excitation during poling but required >50 MV/m if poling was performed without it. In comparison to the (Cd:Zn)S composites, the field strength range suitable for polarization enhancement by optical excitation is narrower for the CdSe hybrid material. The maximum absolute difference was reached using field strength amplitudes between 65 MV/m and 70 MV/m.

**Figure 14:**
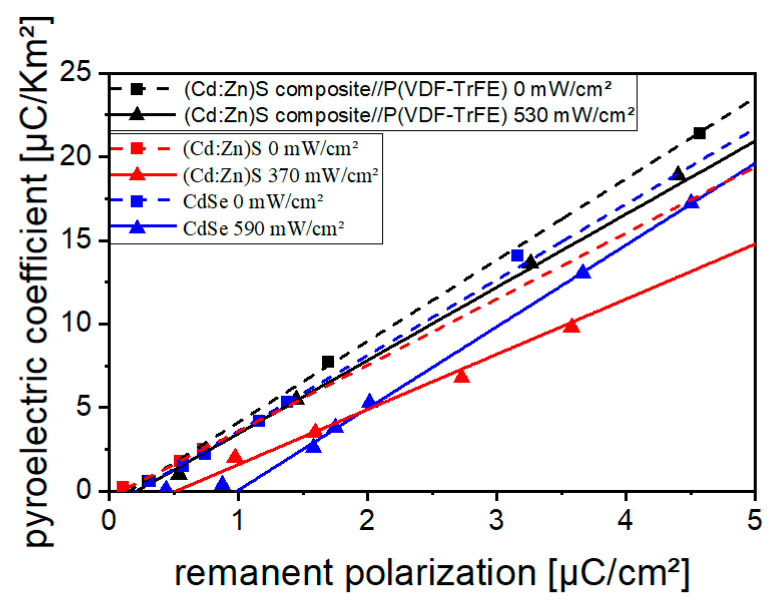
Correlation between *p* and *P_r_* for a composite containing 10 vol% (Cd:Zn)S, a hybrid material with 0.2 Vol% CdSe, as well as a bilayer 10 vol% (Cd:Zn)S composite//P(VDF-TrFE) sample poled without and with optical excitation.

When measuring *P_r_* with the utilized Sawyer–Tower circuit, a distortion by leakage currents is possible. In such cases the measurement of *p* gives a reliable statement on the polarization state of the sample [8]. Due to their higher conductivity under optical excitation (Figure 6), *P_r_* measurements at CdSe and (Cd:Zn)S containing monolayer samples were affected by leakage currents, which can be seen from Figure 14. The distortions are expressed as an offset or a change in the slope of the *p*(*P_r_*) curve. Therefore, *p* should be used to assess polarization change in these cases. Because of their lower electric conductivity (Figure 6), *P_r_* measurements in bilayer samples (and monolayer samples poled without optical excitation) were less effected by leakage currents.

#### 3.4.4. Influence of the Optical Excitation Intensity

**Figure 15:**
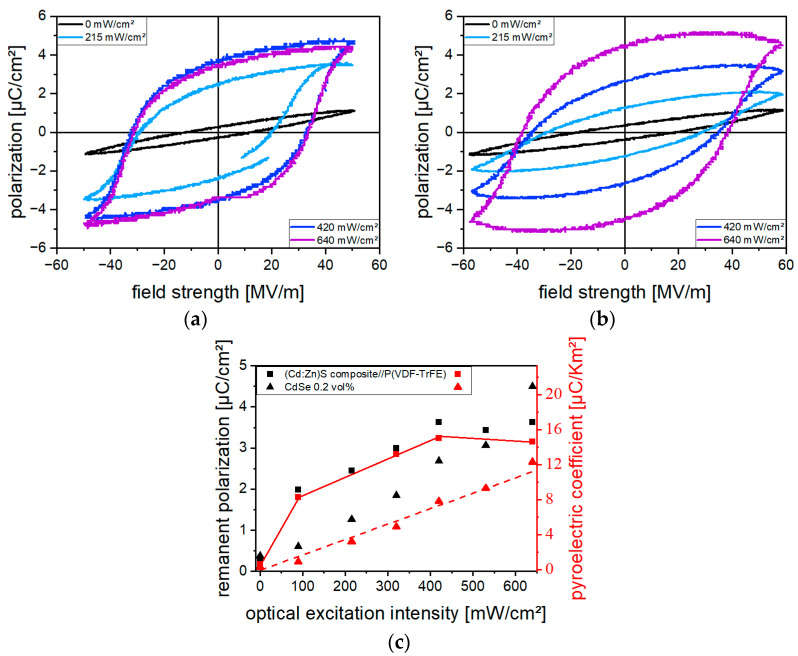
Polarization hysteresis measured at different optical excitation intensities for (**a**) a 0.2 vol% CdSe hybrid material using a field strength amplitude of 57 MV/m and (**b**) a bilayer 10 vol% (Cd:Zn)S composite//P(VDF-TrFE) sample using a field strength amplitude of 50 MV/m. (**c**) Remanent polarization *P_r_* and the pyroelectric coefficient *p* of both samples depending on the optical excitation intensity during poling. Poling was performed with gradually increasing excitation intensities using a poling frequency of 1 Hz.

The influence of optical excitation during poling on polarization hysteresis, *P_r_*, and *p* for a 0.2 vol% CdSe hybrid material and a 10 vol% (Cd:Zn)S composite//P(VDF-TrFE) bilayer sample is shown in Figure 15. For each of the samples, the illumination intensity was increased in steps and the field strength amplitude was kept constant through the poling process. The field strength amplitude for both samples was chosen individually so that a slight polarization occurred even without optical excitation, since the measurements in Figure 13 show that this results in the largest absolute increase in polarization due to optical excitation. The course of the hysteresis of the CdSe hybrid material (Figure 15a), especially of those measured at high illumination intensities, is influenced by leakage currents. This is expressed by the increase in polarization with decreasing field strength. Therefore, *p* is used to assess the polarization state of the sample. In the case of the CdSe hybrid material, a linear increase in *p* occurred from 0.3 µC/(Km^2^) without optical excitation to 12.3 µC/(Km^2^) at 650 mW/cm^2^, and higher intensities led to electrical breakdown. For the bilayer composite, *p* initially increases abruptly at low illumination intensities, then rises linearly to a saturation value of 15 µC/(Km^2^) at 400 mW/cm^2^, before dropping to 14.6 µC/(Km^2^) at the highest intensity used. The results show that polarization of the CdSe hybrid material and the (Cd:Zn)S composite can be controlled by optical excitation.

## 4. Discussion

### 4.1. Electrical Properties

In both the (Cd:Zn)S particle composite and CdSe QDs hybrid material, optical excitation with light of a wavelength of 470 nm led to photoexcitation (Section 3.3 Optical Properties) of the semiconductor filler phase. The thus additionally generated charge carriers resulted in a light-intensity-dependent increase in electrical conductivity (Figure 5). In the case of 0.2 vol% CdSe, the conductivity was raised by a factor of 15 compared to the pure P(VDF-TrFE).

The *I-E* curves of both the 10 vol% (Cd:Zn)S composite and the 0.2 vol% CdSe hybrid material (Figure 5) exhibit nonlinear conduction characteristics, such has been reported for other composite systems of a polymer matrix filled with semiconductor (nano-)particles like CdSe [34,35], CdS [36,37], (Cd:Zn)S [20], or ZnO [19]. Among the rather complex mechanisms causing non-linear conduction behavior in systems of particles and an insulating polymer like P(VDF-TrFE) are charge carrier transport by hopping, tunneling, or thermal activation (or combined mechanisms) over internal interfaces of the particles or particle/particle interfaces [18,38]. The latter case also includes tunneling conduction between particles separated by a thin layer of insulating polymer [19], which is possible from below a minimum distance between individual particles. If paths of directly touching particles occur over the entire sample thickness, these serve as areas of locally increased conductivity. Based on this, the influence of the particle distribution on the electrical conductivity is explained below.

The filler distribution in the 10 vol% (Cd:Zn)S composites was more inhomogeneous compared to the CdSe QDs hybrid material. Areas of locally increased particle concentration were present in the matrix (Figure 3a), especially including agglomerates whose size exceeded the sample thickness. Such uneven particle distribution favors the formation of conduction paths and therefore inhomogeneous conductivity distribution within the composite. Electrical breakdowns occurred along these paths during poling. The presence of these agglomerates could not be prevented even through the use of an ultrasonic sonotrode for particle singulation. Therefore, producing bilayer samples consisting of a layer (Cd:Zn)S composite and a pure P(VDF-TrFE) layer was necessary to ensure breakdown stability. The effect of conduction paths was particularly pronounced in the case of the “single pressed” 10 vol% (Cd:Zn)S composites (Section 3.2). They contained larger agglomerates compared to the other 10 vol% (Cd:Zn)S composites because an ultrasonic bath was used for particle singulation instead of an ultrasonic sonotrode (Figure 3c). In addition, these agglomerates extend over the entire sample thickness due to the single pressing step used for film production. This led to a higher conductivity of the “single pressed” compared to the other (Cd:Zn)S composites, which made it impossible to polarize them at all. It was shown that a comparable increase in electrical conductivity by optical excitation requires a much smaller amount of CdSe QDs than (Cd:Zn)S particles (Figure 5). This is attributed to a finer particle singulation in the CdSe QDs hybrid material. At least a part of the CdSe QDs were present as clusters with diameters < 100 nm (Figure 4) distributed within the P(VDF-TrFE) matrix. If the same volume fraction of filler is assumed, this results in a smaller mean particle spacing than in the (Cd:Zn)S composites. Those had a broad size distribution of primary particles and agglomerates with a mean cluster size of about 800 nm (Figure 3). A smaller mean particle spacing in turn results in better charge carrier transfer between particles [18,19], thus favoring the effectiveness of the QDs for the optically triggered enhancement of conductivity.

**Figure 16:**
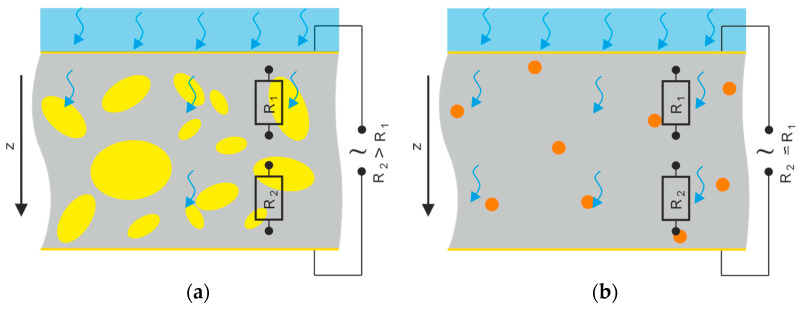
Distribution of light intensity and therefore conductivity over sample thickness direction *z* within the (**a**) 10 vol% (Cd:Zn)S composite and (**b**) 0.2 vol% CdSe hybrid material.

Apart from their finer filler singulation, a more homogeneous conductivity distribution in the CdSe QDs hybrid material is favored by their optical properties. Because of the photoconductive nature of the filler phase, the conductivity of the composite or hybrid material at a sample depth *z* depends on the local light intensity *J*(*z*) as shown in Figure 16. Thus, another conductivity influencing factor is the light intensity distribution occurring within the material (Figure 8), which in turn is affected by the concentration, size, and distribution of the filler. This should result in a uneven conductivity distribution within the 10 vol% (Cd:Zn)S composite (Figure 16a). The illuminated sample side is expected to show high local conductivity compared to the opposite one, which limits the overall conductivity of the sample, at least in the absence of larger agglomerates. This effect is even more pronounced in the bilayer sample with its low-conductive pure P(VDF-TrFE) layer (Figure 6) on the bottom side. Such an inhomogeneous conductivity distribution makes such material systems unsuitable for bifunctional sensor applications with an additional ferroelectric filler phase because parts of the filler would not be supplied with charges and thus could not be polarized [8,16,24]. Furthermore, it is known that such a non-uniform conductivity distribution also leads to the formation of inhomogeneous polarization profiles across the sample cross section [21], which are undesirable for technical applications. In contrast, the smaller filler quantity required for a comparable increase in conductivity in the CdSe hybrid material (Figure 6) led to a homogeneous light intensity distribution within it (Figure 8). This should result in a more homogeneous conductivity distribution (Figure 16b) and therefore polarization profile over the sample thickness, which is further explained in the following section.

Furthermore, the conduction behavior of filler-matrix materials is affected by the properties of the particle/particle and particle/matrix interface. Particle surface properties and their modifications influence their electrical and optical properties [39,40] and the charge carrier transfer between the particle and matrix phase [35]. Although no investigations were carried out in this respect within this work it stands to reason that the functionalization of the CdSe QDs surface with MPA influenced their conduction behavior. Conclusively, a proper tuning of their surface modification would allow an optimization of the QDs regarding the conduction behavior (and singulation as explained in the following section).

### 4.2. Ferroelectric Properties

For both the (Cd:Zn)S- and CdSe-containing material system, an increase in polarization was observed upon poling under optical excitation (Figure 15). A reliable characterization of the polarization state was ensured by measuring the pyroelectric coefficient *p*. The measurement of the remanent polarization *P_r_* was found to be influenced by leakage currents superimposing the displacement current especially for the more conductive (monolayer) samples (Figure 14 and Figure 15c).

Possible explanations for the polarization enhancement due to optical excitation during poling are discussed below.

**Figure 17:**
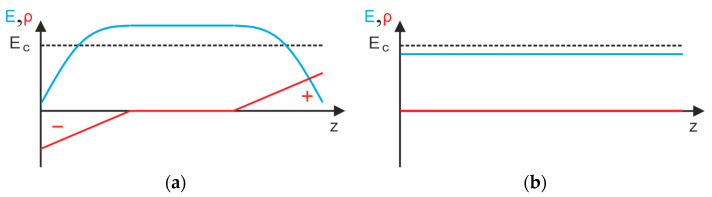
Charge carrier distribution *ρ* and subsequent *E*-field distribution in the 0.2 vol% CdSe hybrid material (**a**) with and (**b**) without optical excitation during poling at an identical average field strength (corresponding to identical poling voltage). The dashed line marks the critical field strength *E_c_* which has to be exceeded to locally polarize the matrix. While no polarization occurs in the unilluminated composite (**b**), dipoles align under illumination (**a**) through distortion of the E-field.

First, it is assumed that the increase in conductivity under illumination (Figure 5 and Figure 6) leads to an increased penetration of charge carriers into the sample during poling. According to Poisson’s equation, the electrical field *E*(*z*,*t*) within the material is distorted by the charge carrier density distribution *ρ*(*z*,*t*) (for the sake of simplification, only uncompensated space charges are considered and the polarization charge density *∂P*(*z*,*t*)/*∂z* is neglected):

(2) $$\varepsilon_{0}\varepsilon_{r}\left(\frac{\partial E\left(z,t\right)}{\partial z}\right)=\rho \left(z,t\right),$$

where *ε_r_* is the dielectric constant, *ε*_0_ the permittivity of the vacuum, *z* the coordinate in thickness direction of the material, and *t* the time during poling. The influence of *ρ*(*z*,*t*) on the polarization behavior of pure P(VDF) and P(VDF-TrFE) [41,42,43], as well as P(VDF-TrFE) composites [21], has already been described in various studies. Based upon this research, it is assumed that the resulting local increase in the field strength in parts of the sample caused by the emerging charge carrier distribution during poling led to the enhanced dipole alignment (i.e., polarization). As an example, an increased charge carrier density in the surface regions of the material due to charge carrier injection would raise the local field strength in deeper sample regions. In Figure 17a, this is shown for the case of the 0.2 vol% CdSe hybrid material. For this, it is assumed that the charge carriers penetrate evenly from both sides due to the homogeneous light intensity and thus conductivity distribution (Figure 16b). In consequence, a symmetrical *E*-field and thus symmetrical polarization distribution in the hybrid material should build up in contrast to the inhomogeneous polarization profiles observed in 10 vol% (Cd:Zn)S composites [21].

A reduced average field strength (equivalent to a reduced poling voltage) was required for the onset of polarization if poling was performed under optical excitation (Figure 13). Charge carrier injection and subsequent *E*-field distortion would also be a possible explanation for this observation as shown comparing Figure 17a,b. Following this thought, a local increase in the field strength (reducing the voltage necessary for poling) could be a possible explanation for the hysteresis effect observed for the measured *I-E* curves (Figure 5). It is possible that slight polarization occurred in parts of the sample due to a local increase in field strength, even at the comparatively low average field strengths used for the measurement. The current through the sample would then be superimposed with a corresponding polarization current. The reason why this effect was especially pronounced for the 10 vol% (Cd:Zn)S composite could be that for these composites the local conductivity enhancement (at least at the illuminated sample side) caused by photoexcitation is comparably high through the almost complete absorption of light at the illuminated sample side (Figure 16a). This in turn would lead to a pronounced local field strength increase.

An increase in polarization was observed by lowering the polarization frequency (Figure 12) or raising the illumination intensity during poling (Figure 15). Both a decreasing frequency (which is equivalent to a raising penetration time for charge carriers before the *E*-field direction is reversed) and higher optical excitation (which leads to increased (photo-)conductivity) should cause an increase in charge charrier injection. This would lead to an enhanced distortion of the *E*-field within the sample and thus to the increased polarization.

For pure P(VDF-TrFE), it is commonly known that compensating charges are required for the stabilization of dipole orientation of the ferroelectric crystals. This in turn is the origin of a stable polarization in the absence of an electrical field (leading to remanent polarization and pyroelectricity). However, the role and origin of the charge carriers is disputed. Ploss et al. [44] achieved stable polarization through poling with ferroelectric but electrical insulating electrodes (which provide no reservoir of free charges). From that they concluded that no charge injection from electrodes is necessary and that compensating localized charges at the sample surface are able to stabilize dipole alignment [44]. Others emphasized the role of injected charges for polarization stabilization. From measurements with and without insulating electrodes [45] and from the time delay between dipole alignment and polarization stabilization [46], it was concluded that charge injection from electrodes and their trapping at the surface of polarized crystals is important for stable polarization [10]. Secondly and referring to that, it could be possible that additional charge carriers (either injected from the electrodes or generated by photoexcitation) influenced the polarization build up and dipole alignment. This could lead to a polarization enhancement under optical excitation in the composites and hybrid materials [21]. It should be stressed that no experimental evidence could be found within the experiments performed in this work which proves that hypothesis.

The polarization enhancement by optical excitation was slightly higher for the 10 vol% (Cd:Zn)S//P(VDF-TrFE) bilayer sample than for the 0.2 CdSe hybrid material (Figure 15). Apart from this, the other results show the advantages of the CdSe hybrid material regarding its optical properties (transparency), as well as the homogeneous distribution of the electrical and presumably ferroelectric properties. In addition to the already highlighted advantages of QDs in the field of optically controlled conductivity and polarization manipulation, two further advantages should be outlined for the sake of completeness. On the one hand, the optical and electrical properties of QDs are adjustable via a large number of parameters [39,47,48,49]. This allows the system to be easily adapted to the light wavelength used as an external trigger. In this context, a response of the system to one or more selected wavelength ranges is possible. This could be performed by the use of multilayer systems with different QDs, for which the observed high transmittance of the QDs system is necessary. On the other hand, the small size of the QDs additive in combination with the control of the system properties by an optical trigger guarantees that switching of electrical and ferroelectric properties can be performed with high spatial resolution. This combination of properties makes a QDs/P(VDF-TrFE) hybrid material attractive for future applications in the field of flexible, high-resolution sensors. In addition to CdSe, other semiconductor materials (e.g., CdS) could be utilized depending on the excitation wavelength and their photoelectric performance. It should be emphasized that the singulation of the QDs in the P(VDF-TrFE) matrix must be further improved to avoid particle accumulations. Approaches to achieve this are already known [50].

## 5. Conclusions

The electrical and polarization properties of mechanically flexible films made of a P(VDF-TrFE) matrix and semiconducting CdSe QDs or (Cd:Zn)S microparticles were investigated with regard to the influence of optical excitation with light of a 470 nm wavelength. In both systems, photoexcitation leads to an increase in electrical conductivity. In addition, electrode poling under optical excitation increased the remanent polarization and the pyroelectric coefficient in both systems. As a result of the finer distribution of the semiconductor phase within the matrix, a much smaller amount of QDs is required for a comparable increase in photoelectric performance. In contrast to the opaque (Cd:Zn)S composite, the CdSe QDs hybrid material is thus transparent, which results in a homogeneous distribution of conductivity and presumably also of polarization across the layer thickness. The combination of its properties makes the hybrid material attractive for applications in the field of flexible, high-resolution sensors. However, further investigations are required to characterize the polarization distribution and the mechanism of polarization enhancement by optical excitation, as well as to optimize the electrical and ferroelectric properties. In addition, the long-term stability of the optoelectrical properties of the QDs and the polarization stability must be clarified as well as possible scaling options for the system. It should also be examined whether CdSe or (Cd:Zn)S can be replaced by a filler material with a lower hazard potential for humans and the environment.

## Data Availability

Data are contained within the article.
